# Evaluation of Off-Hour Emergency Care in Acute Ischemic Stroke: Results from the China National Stroke Registry

**DOI:** 10.1371/journal.pone.0138046

**Published:** 2015-09-17

**Authors:** Guifen Wang, Gaifen Liu, Runhua Zhang, Ruijun Ji, Baoqin Gao, Yilong Wang, Yuesong Pan, Zixiao Li, Yongjun Wang

**Affiliations:** 1 Department of Pediatrics, Beijing Tiantan Hospital, Capital Medical University, Beijing, China; 2 Department of Neurology, Beijing Tiantan Hospital, Capital Medical University, Beijing, China; 3 China National Clinical Research Center for Neurological Diseases, Beijing, China; 4 Center of Stroke, Beijing Institute for Brain Disorders, Beijing, China; 5 Beijing Key Laboratory of Translational Medicine for Cerebrovascular Disease, Beijing, China; St Michael's Hospital, University of Toronto, CANADA

## Abstract

**Background and Purpose:**

The quality of after-hour emergency care of patients with acute ischemic stroke is debatable. We therefore, sought to analyze the performance measures, quality of care and clinical outcomes in these patients admitted during off-hours.

**Methods:**

Our study included 4493 patients from a selected cohort of patients admitted to the hospitals with ischemic stroke in the China National Stroke Registry (CNSR) from September 2007 to August 2008. On-hour presentation was defined as arrival at the emergency department from the scene between 8AM and 5PM from Monday through Friday. Off-hours included the remainder of the on-hours and statutory holidays. The association between off-hour presentation and outcome was analyzed using multivariate logistic-regression models.

**Results:**

Off-hour presentation was identified in 2672 (59.5%) patients with ischemic stroke. Comparison of patients admitted during off-hours with those admitted during on-hours revealed an unadjusted odds ratio of in-hospital mortality of 1.38 (95% confidence interval, 1.04–1.85), which declined to 1.34 (95% confidence interval, 0.93–1.93) after adjusting for patient characteristics (especially, pre-hospital delay). No difference in 30-day mortality, total death or dependence at three, six and 12 months between two groups was observed. No association between off-hour admission and quality of care was found.

**Conclusions:**

In the CNSR database, compared with on-hour patients, off-hour patients with acute ischemic stroke admitted to the emergency departments from scene manifested a higher incidence of in-hospital mortality. However, the difference in incidence and quality of care between the groups disappeared after adjusting for pre-hospital delay and other variables.

## Introduction

Stroke is the second leading cause of death worldwide. From 1990 to 2010, the age-standardized incidence of stroke and burden of stroke increased in both developing and developed countries [1]. Improving quality of care is critical to improve the prognosis after stroke. A higher incidence of mortality and a decreased use of invasive cardiac procedures was noticed in patients admitted with myocardial infarction during weekends, a phenomenon termed the “weekend effect” [2]. Several studies of patients with stroke have reported higher mortality on weekends and weekday nights [3–8]. A recent systematic review and meta-analysis suggests that patients with acute ischemic stroke presenting during off-hours had higher short-term mortality and disability compared with patients presenting during regular hours. The result was robust across subgroups and sensitivity analyses [9]. However, other studies found no significant association between time of admission and stroke care or poor outcomes [10–18]. Since these conflicting results may be based on differences in patient characteristics or differences in the quality of care provided by different medical service systems, prospective clinical registry databases with detailed patient data are needed to establish the role of these factors.

The purpose of this study was to examine whether off-hour presentation was associated with an impact on the quality of care (QOC), mortality or functional outcomes at discharge, one, three, six and 12 months after stroke onset in patients with acute ischemic stroke (AIS) admitted to hospital emergency departments (EDs) in the China National Stroke Registry (CNSR).

## Methods

CNSR is a well-designed, nationwide, prospective cohort database of consecutive stroke patients (older than 18 years) admitted to 132 participating hospitals within 14 days of stroke onset between September 2007 and August 2008 [19]. Acute stroke included ischemic stroke, intracerebral hemorrhage, and subarachnoid hemorrhage. The study was approved by the central Institutional Review Board at Beijing Tiantan Hospital. All patients or their designated proxies provided written informed consent. They consented to participate in this specific study, to have their data entered into the CNSR database and to have their medical data used in future research. These data were fully anonymized and de-identified prior to access.

## Study Population

AIS was diagnosed according to World Health Organization criteria [20] and confirmed using brain computed tomography or magnetic resonance imaging. We chose a cohort of patients admitted to the hospitals with AIS (excluding transient ischemic attack (TIA) and hemorrhagic stroke) that were admitted to the EDs from scene. We excluded subjects admitted to intensive care units (ICUs) and in-patient wards because the patients were those who had initially been admitted to an ICU or in-patient wards for other reasons prior to stroke and who usually had severe medical conditions. We excluded subjects admitted to hospitals from outpatient clinics as they had a milder stroke than other patients and seldom received recombinant tissue plasminogen activator (rTPA, also known as IV rtPA) since thrombolytic therapy were not used in outpatient clinics in Chinese acute care hospitals. We excluded subjects transferred to EDs from other hospitals with probably more severe conditions and poor outcomes.

### Clinical Demographics

We collected the clinical demographics of patients, including age, sex, body-mass index (BMI) at admission, education, vascular risk factors and concomitant diseases (prior stroke/ TIA, smoking, heavy drinking, hypertension, coronary heart disease, atrial fibrillation or flutter, peripheral vascular disease, diabetes mellitus, hyperlipidemia), management (time from onset to admission within three hours (pre-hospital dalay), IV rtPA within three hours of arrival, swallowing assessment, and length of hospitalization), pneumonia and urinary tract infection. The severity of neurologic impairment was evaluated using the National Institutes of Health Stroke Scale (NIHSS) score [21]. The clinical subtypes of ischemic stroke were classified according to Oxfordshire Community Stroke Project (OCSP) criteria. Clinical status on admission was assessed using the modified Rankin Scale (mRS). The patients were admitted to intervention departments of hospital (including stroke unit, neurology ward, neurosurgical intervention, NICU/ICU). CNSR only included grade II hospitals (which serve several communities) and grade III hospitals (designated as teaching hospitals) [22].

### Admission Time

Based on their arrival time at the EDs, we classified patients into on-hour versus off-hour presentation groups. On-hour presentation was defined as arrival at the emergency department between 8AM and 5PM Monday through Friday. Off-hour presentation was defined as arrival during the remainder of the on-hours and statutory holidays.

### Performance Measures

There are currently 13 performance measures for patients with ischemic stroke in China [23]. Except for NIHSS scores at admission, length of stay, and hospital charges, the main performance measures of acute ischemic stroke in China were included in the ten parameters established in the United States. The ten performance indicators [24] quantifying the QOC provided to the AIS admissions are listed in S1 Table. We identified patients as eligible or ineligible for performance indicators according to neurologists treating the patient-diagnosed contraindications.

### Follow-up and Outcomes

At one, three, six, and 12 months, the patient outcomes were evaluated via a telephone follow-up, including death (or mRS = 6) and dependency (defined as mRS = 3 to 5 considered as moderate-to-severe disability). Death was assessed as death due to any cause. Disability was measured by the mRS from 0 to 5. Poor outcomes were defined as death (mRS = 6) or dependency (mRS = 3 to 5). The telephone follow-up was based on a standardized interview protocol.

### Statistical Analysis

Clinical demographics, QOC and outcomes in patients with AIS at emergency admission were compared between on-hours versus off-hours, using *χ* ^*2*^ tests for comparison of categorical variables, Student *t* tests for comparison of the means of continuous variables, and Wilcoxon 2-sample tests for comparison of the medians of continuous variables. The association between off-hour presentation and death and dependency was analyzed in multivariate logistic-regression models, after adjusting for age, sex, NIHSS, smoking, drinking, hypertension, coronary heart disease, atrial fibrillation, diabetes mellitus, dyslipidemia, arrival time within three hours of onset and IV rtPA. All tests were 2-tailed, and a probability value *<* 0.05 was considered significant in univariable and multivariable analyses. Data were analyzed with SAS software, version 9.1.3 (SAS Institute, Inc., Cary, North Carolina).

## Results

### Study Population

**Fig 1:**
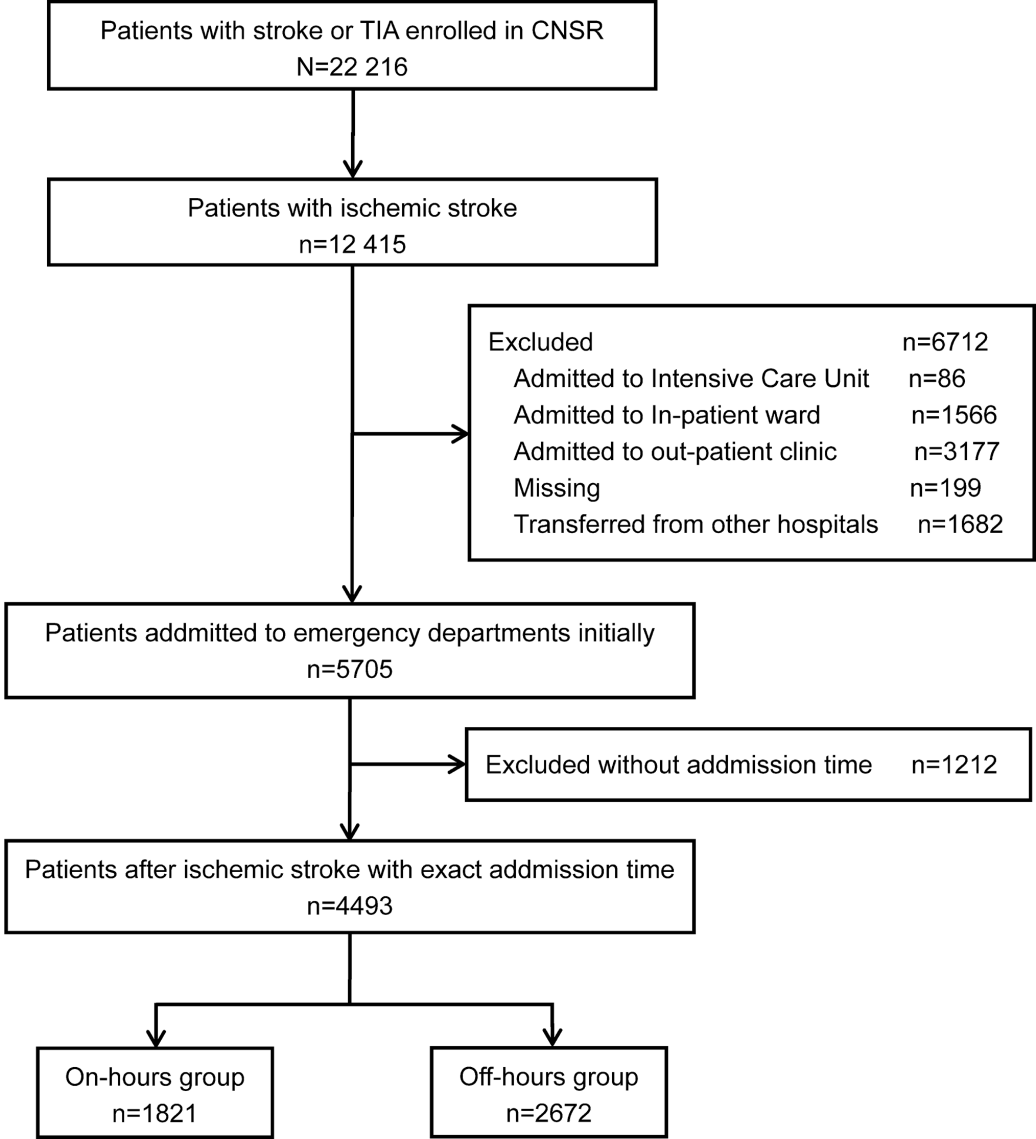
Patient flow diagram. Clinical characteristics of the analyzed population (n = 4493) were compared with patients without exact admission time and excluded from the analyzed patients (n = 1212). No differences in demographic characteristics or risk factor profiles were identified between the two groups, except for the NIHSS score and rtPA within three hours of stroke onset, as shown in S2 Table. Patients with exact admission time had higher NIHSS scores at admission and were more likely to receive rtPA compared with those without admission time.

Of the 22,216 patients with stroke or TIA enrolled in the CNSR, 14,526 were diagnosed with AIS. At one year after stroke onset, follow-up information was available for 12,415 patients with AIS. We excluded subjects that were admitted to ICUs (n = 86), in-patient wards (n = 1566), out-patient clinics (n = 3177) and those transferred to EDs from other hospitals (n = 1682) and missing subjects (n = 199). The final sample included 5705 patients. Patients without an exact time of admission were excluded (n = 1212). Ultimately, 4493 were included in the present study (Fig 1).

### Clinical Demographics

A total of 2672(59.5%) AIS patients were admitted during off-hours. Table 1 shows the demographic profile of AIS patients admitted to the EDs between on-hours and off-hours. The off-hour admission group was slightly younger than the on-hour group (mean age, 66.8 versus 67.5 years, p = 0.056). There were no differences between the two groups in terms of sex, BMI, education, pre-mRS, prior stroke, vascular risk factors, OCSP subtypes, complications, length of hospitalization and intervention departments of hospital. NIHSS scores at admission were similar in the two groups (p = 0.504). Patients who were admitted within three hours of stroke onset constituted a higher proportion in the off-hour group (39.2% versus 33.5%, P < 0.001). No difference was found in the patients with IV rtPA within three hours of stroke onset during off-hours compared with on-hours (3.0% versus 2.8%, P = 0.760). Hospital levels were comparable in the two groups.

**Table 1:** Baseline Characteristics of Patients Admitted to the Emergency Departments between On-hour and Off-hour Presentations after Ischemic Stroke.

Characteristics	Total (n = 4493)	On-hours (n = 1821)	Off-hours (n = 2672)	P Value
Age, y, mean (±SD)	67.1±12.2	67.5±12.3	66.8±12.2	0.056
Sex(% men)	2688 (59.8)	1071 (58.8)	1617 (60.5)	0.253
BMI at admission, kg/m^2^, median(IQR)	24.2(22.0–26.2)	24.2(22.0–26.3)	24.2(22.0–26.2)	0.716
Education (%)				
Higher education	1292 (28.8)	532 (29.3)	760 (28.5)	0.345
Secondary education	1174 (26.2)	455 (25.0)	719 (27.0)	
Elementary education	2017 (45.0)	831 (45.7)	1186 (44.5)	
Prior stroke/TIA (%)	1609 (35.8)	648 (35.6)	961 (36.0)	0.794
Pre-stroke mRS score (%)				
0–1	3346 (74.5)	1344 (73.8)	2002 (74.9)	0.398
2–5	1147 (25.5)	477 (26.2)	670 (25.1)	
Vascular risk factors (%)				
Current smoking	1186 (26.4)	488 (26.8)	698 (26.1)	0.614
Heavy drinking	398 (8.9)	163 (9.0)	235 (8.8)	0.856
Hypertension	2904 (64.6)	1164 (63.9)	1740 (65.1)	0.409
Coronary heart diseases	751 (16.7)	293 (16.1)	458 (17.1)	0.354
Diabetes mellitus	987 (22.0)	411 (22.6)	576 (21.6)	0.421
Hyperlipidemia	521 (11.6)	200 (11.0)	321 (12.0)	0.290
Atrial fibrillation	449 (10.0)	173 (9.5)	276 (10.3)	0.363
Peripheral vascular disease	31 (0.7)	10 (0.6)	21 (0.8)	0.347
NIHSS at admission, median(IQR)	5(3–11)	5(2–10)	5(3–11)	0.145
NIHSS at admission (%)				
≤3	1541 (34.3)	635 (34.9)	906 (33.9)	0.504
>3	2952 (65.7)	1186 (65.1)	1766 (66.1)	
OCSP subtype (%)				
PACI	2452 (57.7)	990 (57.5)	1462 (57.9)	0.404
TACI	508 (12.0)	212 (12.3)	296 (11.7)	
LACI	484 (11.4)	196 (11.4)	288 (11.4)	
POCI	688 (16.2)	283 (16.4)	405 (16.0)	
Time from onset to admission within three hours (%)	1436 (36.9)	528 (33.5)	908 (39.2)	**<0.001**
IV rtPA within 3 hours arrived after stroke onset (%)	130 (2.9)	51 (2.8)	79 (3.0)	0.760
Swallowing assessment (%)	1274 (34.8)	538 (36.2)	736 (33.8)	0.167
Pneumonia (%)	607 (13.5)	238 (13.1)	369 (13.8)	0.476
Urinary tract infection (%)	217 (4.8)	85 (4.7)	132 (4.9)	0.676
Length of hospitalization, d, mean (±SD)	17.3±11.4	17.3±12.2	17.3±10.9	0.954
Admission ward (%)				
Stroke unit	916(20.4)	377(20.7)	539(20.2)	0.980
Neurology ward	3248(72.3)	1311(72.0)	1937(72.5)	
neurosurgical/intervention	15 (0.3)	6 (0.3)	9 (0.3)	
NICU/ICU	314 (7.0)	127 (7.0)	187 (7.0)	
Hospital type (%)				
Grade III	3604 (80.2)	1454 (79.8)	2150 (78.5)	0.610
Grade II	889 (19.8)	367 (20.2)	522 (21.5)	

Abbreviations: SD, standard deviation; BMI, body-mass index; IQR, interquartile range; TIA, transient ischemic attack; mRS, modified Rankin Scale; NIHSS, National Institutes of Health Stroke Scale; OCSP, Oxfordshire Community Stroke Project criteria; PACI, partial anterior circulation infarct; TACI, total anterior circulation infarct; LACI, lacunar infarct; POCI, posterior circulation infarct; IV rtPA, intravenous recombinant tissue plasminogen activator.

### Performance Measures

No significant difference was observed in the quality of care provided to patients admitted to hospital during off-hours and on-hours (Table 2). The proportion of patients who received stroke health education during off-hours was slightly higher than those during on-hours (67.4% vs 64.4% P = 0.070), similar to the proportion with smoking cessation (61.6% vs 57.6%) and rehabilitation assessment (50.6% vs 48.3%). However, DVT prophylaxis rates, rates of thrombolytic therapy and dysphagia screening rates were slightly lower during off-hour admissions (40.5% vs 41.9%, 14.1% versus 15.6%, 46.2% vs 48.6%). A slightly higher number of patients were discharged on anticoagulation for atrial fibrillation during off-hour admissions (23.1% vs 18.8%), similar to those discharged on cholesterol-reducing medication (38.1% vs 37.3%) and antithrombotic therapy (66.9% vs 64.8%).

**Table 2:** Quality of Care Indicators and Off-hour vs On-hour Presentation after Ischemic Stroke.

Performance measures	Total(n/N,%)	On-hours (n/N,%)	Off-hours (n/N,%)	P Value
Total number	4493	1821	2672	-
1. DVT prophylaxis	757/1842(41.1)	309/737(41.9)	448/1105(40.5)	0.554
2. Discharged on antithrombotic therapy	2894/4383(66.0)	1151/1777(64.8)	1743/2606(66.9)	0.147
3. Discharged on Anticoagulation for patients with AF	109/510(21.4)	39/207(18.8)	70/303(23.1)	0.826
4. Thrombolytic therapy administered	98/671(14.6)	37/238(15.6)	61/433(14.1)	0.609
5. Antithrombotic therapy by the end of hospital Day 2	3477/4325(80.4)	1415/1743(81.2)	2061/2557(80.6)	0.635
6. Discharged on cholesterol-reducing medication	1164/3084(37.7)	459/1230(37.3)	705/1853(38.1)	0.670
7. Dysphagia screening	1626/3448(47.2)	682/1403(48.6)	944/2045(46.2)	0.157
8. Stroke education	2974/4493(66.2)	1172/1821(64.4)	1802/2672(67.4)	0.070
9. Smoking cessation	1056/1761(60.0)	415/721(57.6)	641/1040(61.6)	0.129
10. Assessed for rehabilitation	2233/4493(49.7)	880/1821(48.3)	1353/2672(50.6)	0.128

Abbreviations: DVT, deep vein thrombosis; AF, atrial fibrillation; (n/N, %): n, of adherence; N, of eligible; %, Adherence rate.

Improved organization of stroke services may have a huge impact on stroke outcomes. We therefore, assessed the off-hour effect on quality of care indices between grade II and grade III hospitals (S3 Table) and found no difference between two groups.

### Outcomes

Table 3 shows that the in-hospital mortality rate was 5.4% for off-hour presentation compared with 4.0% for on-hour presentation (P = 0.028). The multivariate logistic-regression analysis suggested that the in-hospital mortality during off-hour admissions decreased the odds ratio to 1.34 (95%CI, 0.93–1.93; P = 0.118) compared with on-hour admissions after adjusting for patient characteristics, in particular, pre-hospital delay (Table 4). There were no differences in 30-day mortality, total death or dependence at three, six and 12 months between two groups.

**Table 3:** Comparison of Patient Outcomes between On-hour and Off-hour Presentations after Ischemic Stroke.

Outcomes	Total (n = 4493)	On-hours (n = 1821)	Off-hours (n = 2672)	P Value
Discharge death (%)	216 (4.8)	72 (4.0)	144 (5.4)	**0.028**
30-day mortality (%)	269 (6.0)	103 (5.7)	166 (6.2)	0.440
Death or dependency at 3 mo (%)	1765 (39.4)	719 (39.7)	1046 (39.3)	0.772
Death at 3 mo (%)	430 (9.6)	172 (9.5)	258 (9.7)	0.834
Death or dependency at 6 mo (%)	1752 (39.1)	714 (39.4)	1038 (39.0)	0.760
Death at 6 mo (%)	548 (12.2)	225 (12.4)	323 (12.1)	0.765
Death or dependency at 12 mo (%)	1734 (38.7)	714 (39.4)	1020 (38.3)	0.446
Death at 12 mo (%)	700 (15.6)	290 (16.0)	410 (15.4)	0.597

**Table 4:** Unadjusted and Adjusted Odds Ratios of Poor Outcomes in Ischemic Stroke Patients: Off-hour versus On-hour Presentations.

Outcomes	UnadjustedOR (95% CI)	P Value	AdjustedOR (95% CI)	P Value
Discharge death	1.38 (1.04–1.85)	**0.028**	1.34(0.93–1.93)	0.118
30-day mortality	1.11 (0.86–1.42)	0.440	1.23(0.88–1.71)	0.218
Death or dependency at 3 mo	0.98 (0.87–1.11)	0.772	1.06(0.89–1.26)	0.530
Death at 3 mo	1.02 (0.83–1.25)	0.834	1.06(0.81–1.39)	0.650
Death or dependency at 6 mo	0.98 (0.87–1.11)	0.760	1.05(0.88–1.24)	0.612
Death at 6 mo	0.97 (0.81–1.16)	0.765	0.97(0.76–1.24)	0.802
Death or dependency at 12 mo	0.95 (0.84–1.08)	0.446	1.00(0.84–1.19)	0.983
Death at 12 mo	0.96 (0.81–1.13)	0.597	0.94(0.75–1.17)	0.581

Abbreviations: OR, odds ratio; CI, confidence interval. Adjusted for age, sex, National Institutes of Health Stroke Scale, smoking, drinking, hypertension, coronary heart disease, atrial fibrillation, diabetes mellitus, dyslipidemia, IV rtPA, and time from onset to admission within three hours (pre-hospital delay).

## Discussion

In this study, we investigated the association between off-hour presentation and outcomes in patients with acute ischemic stroke. We found that in-hospital mortality was slightly higher in patients with ischemic stroke admitted to hospital emergency departments during off-hours compared with on-hours (5.4% vs 4.0%, p = 0.028). After adjustment for potential confounding variables, no association between off-hour presentation and increased mortality was observed. Further, no significant differences were detected in the quality of stroke care provided during off-hour admissions compared with on-hours.

Previous studies reported a “weekend effect” in that stroke patients admitted on weekends or weekday nights had worse outcomes including increased seven-day mortality, in-hospital mortality, 30-day and 90-day mortality, 90-day mRS or decreased discharge to usual place of residence [3–8]. The effect was attributed to suboptimal quality of care (care effect) or more severe disease (patient effect). An Australian study of 539,122 patients admitted to emergency departments of all 501 hospitals in New South Wales between 2000 and 2007 suggested that higher in-hospital mortality of stroke on weekend admissions was due to reduced quality of care and different patient cohorts [25]. Another study examined data from 82,219 ischemic stroke admissions to 115 Dutch hospitals between 2000 and 2004 and reported increased deaths from midnight to 7:00 and decreased death incidence from 14:00 to 18:00 compared with admissions at 8:00. The protective effect during shift changes suggested that increasing the number of staff available improved quality of care and outcome [7]. A Swedish study found that the “weekend effect” decreased with time (with increased quality of stroke care) [3]. In addition, the “weekend effect” was reduced or eliminated by participating in stroke clinical improvement programs such as Get With the Guidelines (GWTG) [5] or using comprehensive stroke centers [14]. However, these studies were correlated with large administrative databases and retrospective studies, with limited clinical data for outcomes research, such as severity of stroke. Recent single- or multi-center prospective studies found no “weekend effect” when adjusted for NIHSS score, pre-hospital delay or other factors [15,16]. In our cohort of patients admitted to the emergency departments with AIS in the CNSR, no significant differences were found in demographics and severity of stoke (expressed by NIHSS score) except for pre-hospital delay.

A recent study of stroke care in the Denmark (using the Danish Stroke Registry) found that patients admitted during off-hours showed a lower degree of compliance with 8 out of 10 performance measures between 2003 and 2011 [26]. In contrast, our study showed that patients admitted during off-hours did not receive a poorer quality of care between 2007 and 2008 in the CNSR database. Differences in study design may account for the differences in the Danish study and our study. The Danish study was limited to admissions with a first-ever acute stroke in university or non-university Danish hospitals, whereas our study was limited to patients admitted with ischemic stroke at the Grade II or III hospital (more likely to be tertiary care hospitals in urban areas) emergency departments. Further, we included holiday admissions in the off-hour cohort, unlike the Danish study, which reported that the admission time-related differences in care were substantially reduced over time (after implementation of a national systematic quality improvement program).

Interestingly, we found that the off-hour group may be more likely to receive stroke health education compared with on-hour group (67.4% vs 64.4%, P = 0.070) in our study. One possible explanation is that patients admitted to the hospital during off-hours were customarily considered to have more severe stroke by physicians [27]. Therefore, doctors spend more time explaining the condition and provide more stroke prevention education. Patients were also more likely to be with family members during off-hours. Therefore, family members may pay more attention to the patient’s condition and be more likely to receive prevention information of stroke. In addition, thrombolysis between the two groups was similar. However, the rate of arrival time from stroke onset to admission within three hours in the off-hour presentation group was higher than in on-hour group (P < 0.001) suggested that the thrombolytic rate in off-hour presentation in China may be relatively lower compared with on-hour presentation. Approximately 2% of all patients with AIS from the CNSR [28] and in our study 2.5% of all patients with AIS admitted to the EDs from scene received thrombolysis with IV rtPA within three hours of admission, which was lower than the thrombolysis in United States and Germany [13, 29]. The finding suggests that efforts should be increased to improve the rate of thrombolysis by optimizing the process of thrombolysis during weekends or off-hours. Although we found no significant differences of quality of stroke care in the CNSR database between off-hour and on-hour admissions, it is possible that these performance measures do not reflect the overall quality of stroke care provided by acute hospitals due to lack of utilization of clinical interventions such as carotid endarterectomy, hypertension control, and use of stroke units. Thus, our findings do not negate the need for initiatives aimed at improving staffing and access to resources on off-hour presentations.

In addition, a recent systematic review and meta-analysis suggested that off-hour presentation of patients with acute ischemic stroke was associated with significantly higher short-term mortality (OR, 1.11, 95% CI 1.06–1.17) [9]. Comparison of off-hours versus regular hours was categorized as: 1) weekend and night vs. weekday regular hours, 2) weekend vs. weekday, or 3) night vs. day. Because the study had a high heterogeneity (I^2^ > 80%), the subgroup analyses showed no higher short-term mortality between weekend and night vs. weekday regular hour (OR, 1.11, 95% CI 0.99–1.25), similar to our study. The finding suggests that the differences in mortality or disability between off- and on-hours are attributed to differences in population demographics and clinical characteristics or healthcare services in different countries.

Currently, the level of quality of stroke care in multicenter clinical registry or national database in China is unknown. However, a recent Chinese study found that stroke patients admitted to 109 grade III class A hospitals between 2007 and 2010 showed a declined in-hospital mortality (from 3.2% to 2.3%), indicating improvements in care and prevention of acute stroke [30]. The in-hospital mortality in this study was nearly equal to the data from the China National Stroke Registry [31], but lower than that of Germany and the United States.

In the CNSR, most stroke patients were admitted into stroke units and neurology wards. The stroke units offer acute medical treatment and multidisciplinary care under a standardized protocol for diagnosis, treatment and rehabilitation of patients with acute stroke. The neurology wards serve patients with diseases of the nervous system and not stroke alone. In our study, we found no off-hour effect on admission ward. Additionally, no off-hour impact was seen on quality of stroke care between Grade II and Grade III. Our study suggests that patients in the CNSR database with acute ischemic stroke admitted to hospital EDs from scene received a consistent quality of care round the clock.

The strength of our study is related to the use of a prospective database that was adjusted for the clinical state at admission such as pre-hospital delay, and severity of stroke. Our database records the date and time of admission, and thus provides an opportunity to investigate the quality of care and poor outcome on weekends, weekday nights and statutory holidays when staffing and available resources are relatively reduced.

There are several limitations in our study. First, the admission of 1212 (21.2%) patients first admitted to the EDs was not accurately recorded, leading to possible bias. However, baseline characteristics of the groups with known and unknown admission time were comparable except for NIHSS score at admission, which was higher in the former group. The use of rt-PA within three hours after onset showed a similar trend. Second, the hospitals that participated in the CNSR were voluntary and focused on research, and were more likely to be tertiary care hospitals in urban areas and therefore endowed with greater resources and stroke specialties than smaller hospitals in rural areas. Thus, the general conclusions of this study may not apply to other types of institutions or countries with different health care systems.

## Conclusions

In the CNSR database from 2007 to 2008, no associations were seen between hospital admission time and mortality in patients with acute ischemic stroke first admitted to the EDs even after adjustment for pre-hospital delay and other parameters. No significant difference was found in quality-of-care indicators in off-hour presentations compared with on-hour cases.

## Supporting Information

S1 Table Current Stroke Performance Measures as Endorsed by the Major Stroke Quality Improvement Organizations in the United States. Abbreviations: DVT, deep vein thrombosis; AF, atrial fibrillation; EMS, emergency medical services.
(PDF)

S2 Table Patient Profile with or without Admission Time after Ischemic Stroke. Abbreviations: AT, admission time; SD, standard deviation; IQR, interquartile range; NIHSS, National Institutes of Health Stroke Scale; IV rtPA, intravenous recombinant tissue-type plasminogen activator.
(PDF)

S3 Table Off-hour Effect on Quality-of-Care Indices in Grade II and Grade III Hospitals. Abbreviations: DVT, deep vein thrombosis; AF, atrial fibrillation; (n, %): n, of adherence; %, Adherence rate.
(PDF)
