# Supplementary material for: Evaluation of Off-Hour Emergency Care in Acute Ischemic Stroke: Results from the China National Stroke Registry
Source: PLoS One. 2015 Sep 17;10(9):e0138046. doi: 10.1371/journal.pone.0138046 (PMC4574931; doi:10.1371/journal.pone.0138046)
Supplement: S1 Table — Abbreviations: DVT, deep vein thrombosis; AF, atrial fibrillation; EMS, emergency medical services. (PDF) [file pone.0138046.s001.pdf]

S1 Table. Current Stroke Performance Measures as Endorsed by the Major Stroke Quality Improvement Organizations in the United States.

**Indicator**	**Definition**
1. DVT prophylaxis	Nonambulatory patients should start receiving DVT prophylaxis by end of hospital Day 2
2. Discharged on antithrombotic therapy	Patients prescribed antithrombotic therapy (aspirin, clopidogrel, dipyridamole, or oral anticoagulants) at discharge
3. Discharge on Anticoagulation for patients with AF	Patients with AF discharged on anticoagulation
4. Thrombolytic therapy administered	Patients with acute ischemic stroke who arrive at the hospital within 120 minutes (2 hours) of time last known well and for whom intravenous tissue plasminogen activator was initiated at this hospital within 180 minutes (3 hours) of last known well
5. Antithrombotic therapy by the end of hospital Day 2	Patients who receive antithrombotic therapy by the end of hospital Day 2
6. Discharged on cholesterol-reducing medication	Patients with LDL > 100mg/dl, or LDL not measured, or on cholesterol-reducer before admission, who are discharged on cholesterol-reducing drugs

7. Dysphagia screening	Patients who undergo screening for dysphagia with a simple valid bedside testing protocol before being given any food, fluids, or medication by mouth
8. Stroke education	Patients or their caregivers who were given education or educational materials during the hospital stay addressing all of the following: personal risk factors for stroke, stroke warning signs, activation of EMS, need for follow-up after discharge, and medications prescribed
9. Smoking cessation	Patients with a history of smoking cigarettes who are, or whose caregivers are, given smoking cessation advice or counseling during hospital stay; a smoker is defined as someone who has smoked cigarettes anytime during the year before hospitalization
10. Assessed for rehabilitation	Patients who are assessed for rehabilitation

Abbreviations: DVT, deep vein thrombosis; AF, atrial fibrillation; EMS, emergency medical services.
