# Supplementary material for: Evaluation of Off-Hour Emergency Care in Acute Ischemic Stroke: Results from the China National Stroke Registry
Source: PLoS One. 2015 Sep 17;10(9):e0138046. doi: 10.1371/journal.pone.0138046 (PMC4574931; doi:10.1371/journal.pone.0138046)
Supplement: S2 Table — Abbreviations: AT, admission time; SD, standard deviation; IQR, interquartile range; NIHSS, National Institutes of Health Stroke Scale; IV rtPA, intravenous recombinant tissue-type plasminogen activator. (PDF) [file pone.0138046.s002.pdf]

S2 Table. Patient Profile with or without Admission Time after Ischemic Stroke.

**Characteristics**	**Total (n=5705)**	**With AT （n=4493）**	**Without AT （n=1212）**	**P Value**
Age, y, Mean (±SD)	67.2±12.2	67.1±12.2	67.7±11.9	0.102
Sex (% men)	3399 (59.6)	2688 (59.8)	711 (58.7)	0.464
NIHSS score at admission, median (IQR)	5(2-10)	5(3-11)	5(2-10)	**<0.001**
Smoking (%)	1478 (25.9)	1186 (26.4)	292 (24.1)	0.104
Hypertension (%)	3721 (65.2)	2905 (64.7)	816 (67.3)	0.083
Diabetes mellitus (%)	1280 (22.4)	987 (22.0)	293 (24.2)	0.102
Atrial fibrillation (%)	559 (9.8)	449 (10.0)	110 (9.1)	0.340
Dyslipidemia (%)	654 (11.5)	521 (11.6)	133 (11.0)	0.546
Heart failure (%)	129 (2.3)	98 (2.2)	31 (2.6)	0.434
Coronary heart disease (%)	958 (16.8)	751 (16.7)	207 (17.1)	0.763
IV rtPA within 3 hours arrived after stroke onset (%)	141 (2.5)	130 (2.9)	11 (0.9)	**<0.001**

Abbreviations: AT, admission time; SD, standard deviation; IQR, interquartile range; NIHSS, National Institutes of Health Stroke Scale; IV rtPA, intravenous recombinant tissue-type plasminogen activator.
