# Supplementary material for: Evaluation of Off-Hour Emergency Care in Acute Ischemic Stroke: Results from the China National Stroke Registry
Source: PLoS One. 2015 Sep 17;10(9):e0138046. doi: 10.1371/journal.pone.0138046 (PMC4574931; doi:10.1371/journal.pone.0138046)
Supplement: S3 Table — Abbreviations: DVT, deep vein thrombosis; AF, atrial fibrillation; (n, %): n, of adherence; %, Adherence rate. (PDF) [file pone.0138046.s003.pdf]

S3 Table. Off-hour Effect on Quality-of-Care Indices in Grade II and Grade III Hospitals.

Hospital type (total number)	Grade II (n=889)			Grade III (n=3604)		
**Performance measures**	**On-hours (n,%)**	**Off-hours (n,%)**	**P value**	**On-hours (n,%)**	**Off-hours (n,%)**	**P value**
Total number	367(41.3)	522(58.7)	-	1454(40.3)	2150(59.7)	-
1. DVT prophylaxis	54(33.5)	80(35.4)	0.705	255(44.3)	368(41.8)	0.365
2. Discharged on antithrombotic therapy	241(69.1)	359(71.5)	0.439	910(63.7)	1384(65.8)	0.209
3. Discharged on Anticoagulation for patients with AF	9(18.8)	16(24.6)	0.458	30(18.9)	54(22.7)	0.361
4. Thrombolytic therapy administered	10(17.9)	13(15.9)	0.757	27(14.8)	48(13.7)	0.715
5. Antithrombotic therapy by the end of hospital Day 2	298(86.6)	410(84.0)	0.298	1117(79.8)	1651(79.8)	0.974

6. Discharged on cholesterol-reducing medication	98(41.0)	132(39.6)	0.743	361(36.4)	573(37.7)	0.508
7. Dysphagia screening	130(48.0)	179(45.1)	0.463	552(48.8)	765(46.4)	0.224
8. Stroke education	270(73.6)	408(78.2)	0.282	902(62.0)	1394(64.8)	0.128
9. Smoking cessation	95(71.4)	143(76.1)	0.526	320(54.4)	498(58.5)	0.146
10. Assessed for rehabilitation	194(52.9)	296(56.7)	0.257	686(47.2)	1057(49.2)	0.243

Abbreviations: DVT, deep vein thrombosis; AF, atrial fibrillation; (n, %): n, of adherence; %, Adherence rate.
